# Oncolytic Newcastle Disease Virus Co-Delivered with Modified PLGA Nanoparticles Encapsulating Temozolomide against Glioblastoma Cells: Developing an Effective Treatment Strategy

**DOI:** 10.3390/molecules27185757

**Published:** 2022-09-06

**Authors:** Zahraa A. Kadhim, Ghassan M. Sulaiman, Ahmed M. Al-Shammari, Riaz A. Khan, Osamah Al Rugaie, Hamdoon A. Mohammed

**Affiliations:** 1 Division of Biotechnology, Department of Applied Sciences, University of Technology, Baghdad 10066, Iraq; 2 Department of Experimental Therapy, Iraqi Centre for Cancer and Medical Genetics Research, Mustansiriyah University, Baghdad 14022, Iraq; 3 Department of Medicinal Chemistry and Pharmacognosy, College of Pharmacy, Qassim University, Qassim 51452, Saudi Arabia; 4 Department of Basic Medical Sciences, College of Medicine and Medical Sciences, Qassim University, Unaizah 51911, Saudi Arabia; 5 Department of Pharmacognosy and Medicinal Plants, Faculty of Pharmacy, Al-Azhar University, Cairo 11371, Egypt

**Keywords:** temozolomide (TMZ), polylactic-co-glycolic acid (PLGA), PVA, Newcastle disease virus (NDV), glioblastoma, AMGM5 cells, REF cells, cytotoxicity, combination therapy

## Abstract

Glioblastoma multiforme (GBM) is considered to be one of the most serious version of primary malignant tumors. Temozolomide (TMZ), an anti-cancer drug, is the most common chemotherapeutic agent used for patients suffering from GBM. However, due to its inherent instability, short biological half-life, and dose-limiting characteristics, alternatives to TMZ have been sought. In this study, the TMZ-loaded PLGA nanoparticles were prepared by employing the emulsion solvent evaporation technique. The prepared TMZ-PLGA-NPs were characterized using FT-IR, zeta potential analyses, XRD pattern, particle size estimation, TEM, and FE-SEM observations. The virotherapy, being safe, selective, and effective in combating cancer, was employed, and TMZ-PLGA-NPs and oncolytic Newcastle Disease Virus (NDV) were co-administered for the purpose. An AMHA1-attenuated strain of NDV was propagated in chicken embryos, and the virus was titrated in Vero-slammed cells to determine the infective dose. The in vitro cytotoxic effects of the TMZ, NDV, and the TMZ-PLGA-NPs against the human glioblastoma cancer cell line, AMGM5, and the normal cell line of rat embryo fibroblasts (REFs) were evaluated. The synergistic effects of the nano-formulation and viral strain combined therapy was observed on the cell lines in MTT viability assays, together with the Chou–Talalay tests. The outcomes of the in vitro investigation revealed that the drug combinations of NDV and TMZ, as well as NDV and TMZ-PLGA-NPs exerted the synergistic enhancements of the antitumor activity on the AMGM5 cell lines. The effectiveness of both the mono, and combined treatments on the capability of AMGM5 cells to form colonies were also examined with crystal violet dyeing tests. The morphological features, and apoptotic reactions of the treated cells were investigated by utilizing the phase-contrast inverted microscopic examinations, and acridine orange/propidium iodide double-staining tests. Based on the current findings, the potential for the use of TMZ and NDV as part of a combination treatment of GBM is significant, and may work for patients suffering from GBM.

## 1. Introduction

Cancer, a fatal disease, results from unregulated cells division caused by both hereditary and environmental factors. Despite the contemporary advances in detection, control, and treatment, cancer continues to impact millions of people throughout the world, and remains a leading cause of mortality [1]. The International Agency for Research on Cancer (IARC), the World Health Organization’s (WHO) specialized cancer agency, published a cancer information sheet for Iraq in 2020, which was based on data from 2018. According to the datasheet, there were 25,320 new cases, and 14,523 fatalities reported in the country during the period. Lung cancer (2066 deaths), breast cancer (1727 deaths), leukemia (1327 deaths), brain and central nervous system malignancies (1085 deaths), and stomach cancer (750 deaths) were the commonly diagnosed cancer fatalities. According to 2020 statistics on cancer in the country, it was found that the incident rate of brain cancer was at 4.7%, with 1600 new cases out of the overall number of the new cancer cases reported from the country. The brain tumor ranked 4th in number of mortality rates, with almost 7% of overall cancer-related deaths falling in this category [2,3,4]. 

Glioblastoma multiforme (GBM; WHO grade IV glioblastoma) is the most prevalent malignant glioma, with a median survival time of just 14.6 months, when treated with standard therapy, and this includes maximally safe surgical resection, followed by radiation therapy with concurrent and adjuvant temozolomide (TMZ) treatment [5]. 

**Figure 1:**
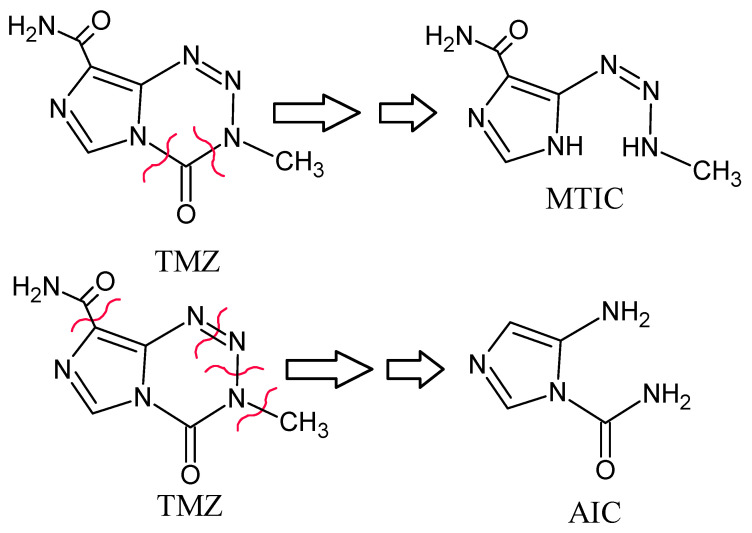
Structures of TMZ, its active constituent MTIC, and the metabolite AIC; produced after biodegradation of the TMZ at the plausible structural sites indicated by the red cuttings.

The TMZ, considered as the gold standard therapy for GBM, is a DNA methylating and cross-linking agent, which when administered at physiological pH, produces MTIC (5-(3-methyltriazen-1-yl)-imidazole-4-carboxamide) as the active constituents, and AIC (5-amino-imidiazole-4-carboxoamide) as the metabolite (Figure 1). It has some drawbacks, including limited bioavailability, and severe toxicity, both of which restrict its pharmacological benefits [6]. The TMZ is the most common oral formulation after surgical resection of GBM. The TMZ is biologically an unstable molecule, with a short half-life, and has restricted dose regimen. Therefore, new strategies to increase the drug’s stability, bioavailability, and biological half-life, to protect the drug from systemic degradation/modification, as well as to enhance its delivery to the cancer cells are much needed. As a result, encapsulating the TMZ in a biodegradable material as a nano-carrier appears to be a plausible way of overcoming the aforementioned deficiencies, and boost the chemotherapeutic efficacy of the drug, to provide safe delivery, and increase the drug’s accumulation at the target site [7].

Nanotechnology, employing methodologies to prepare materials, and devices with unique characteristics and sizes, often in the range of 1 to 100 nanometers, seems to be a viable option to introduce new drugs in the nanomedicine domain [8]. Polymeric nanoparticles have shown to increase the drug’s bioavailability to the delivered cancer cells, while also overcoming the multidrug resistance, thereby providing safe delivery through systemic circulation, and also through direct oral delivery, if adopted [9]. Poly(D,L-lactide-co-glycolide) (PLGA), a biodegradable and biocompatible polymer, is among the most commonly used material for the purpose. PLGA is an US-FDA-approved, nanoscale moldable material that provides adjustable physicochemical properties according to the delivery needs [10]. Polyvinyl alcohol (PVA), also a chemically stable, and biocompatible material, has several purposes in the nano-technical realm dealing with the stabilization, and drug delivery of the intended therapeutic agent in nano-form preparations. Owing to the superior mechanical properties, and tunable characteristics of these materials, the PLGA and PVA are frequently employed in several biomedical applications, including for the drug delivery purposes. PVA, which has, comparatively to the PLGA and other polymers, a slightly lower level of integration into the living tissue due to its low biodegradability, and biocompatibility, has been less frequently utilized. However, it provides better stabilization to the nano-colloids used in drug delivery [11]. Notwithstanding, the cancer has been treated with classical therapeutic procedures, such as, surgery, chemotherapy, immunotherapy, hormonal, and other targeted therapy. Also, in parallel, new protocols have been examined to treat cancers such as gene therapy [12], nano-therapy [13], and viral therapies [14]. The viral therapy’s background dates back to 1912, and the results obtained were confounding [15]. However, continuous improvements and modern approaches have altered the situation, and viral therapy has now been part of established treatment protocols for cancer therapy. Among several techniques, the oncolytic viruses (OVs) have gained significant importance as part of the modern-day approaches in cancer containments [16,17]. Importantly, the OVs can only be spread in tumor cells, and they do not affect any normal cell [18]. On the contrary, the majority of the marketed chemotherapeutic agents are difficult to safe deliver, and are harmful to healthy tissues, thereupon producing harmful side effects [19]. In this context for OVs, the virotherapy has emerged as a viable option, since the viral replication allows for the injected dose to be continually amplified until the immune system intervenes. These viral agents are also capable of penetrating, and destroying the cancer cells while causing no harm to the neighboring normal cells. These viral entities are also genetically stable and safe, with few adverse effects [20,21,22]. The OVs were the first to be used in treatment of specific types of cancers approximately five decades ago, and the NDV, a naturally occurring virus, has been utilized in early-phase investigations for the treatment of glioma [23,24]. NDV, a poultry virus, is non-pathogenic to mammals, including humans, and belongs to the *Paramyxoviridae* family, and to genus *Othoavulavirus* [25].

The current proposition was aimed to prepare PLGA nanoparticles, TMZ-PLGA-NPs, for loading of the anticancer drug, TMZ, for glial cell targeting. The produced nanoparticles were intended to be able to cross through the blood–brain barrier (BBB), and reach the target cells, thereby demonstrating the enhanced bioavailability through improved penetrability of the nano-carrier into the malignant cells. The therapeutic performance indices of the pure, free, and the encapsulated drugs were also assessed. The plan of work was to demonstrate TMZ-PLGA-NPs as a useful modality for treatment of gliomas in combination with the NDV, to break the cancer resistance, and treat the diseased cancer condition, as part of combination therapy with the NDV mediation on the AMGM5 human glioblastoma cancer cell lines.

## 2. Results and Discussion

### 2.1. Preparation of TMZ-PLGA Nanoparticles

**Figure 2:**
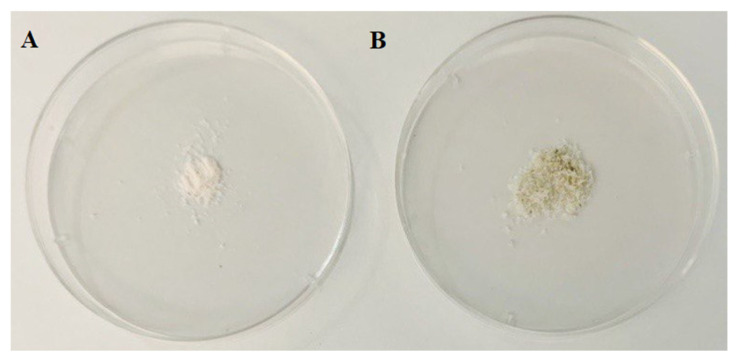
Photographs showing: (**A**) pure, free TMZ powder; (**B**) drug loaded TMZ-PLGA-NPs.

Successful preparation of TMZ-PLGA-NPs, (Figure 2), was achieved through the application of the emulsion solvent evaporation technique with the utilization of PLGA and PVA polymers. With the formulation system design, it was a priority to ensure easy release of the loaded drug from its encapsulating material, e.g., PLGA. This was due to the fact that any restriction on the release of the drug may lead to undesired interference with its bioavailability, and cause impairment of its therapeutic efficacy [26]. The low water solubility, and inherent instability of the TMZ in water, reportedly, imparted the preparation of TMZ injections as extremely problematic. Although the TMZ, to a certain extent, is soluble and stable in acidic circumstances after oral administration, and because of its low half-life of only 1.8 h, is quickly removed from the systemic circulation [27,28]. Furthermore, in the bloodstream, the TMZ degrades to MTIC, which does not cross the BBB, made the PLGA encapsulation of the drug inevitable. The PLGA based nano-carrier, through its design characteristics, and size control, have capability to improve the solubility, and bioavailability of the TMZ. The nano-carrier also provided prolonged release of the TMZ, thereby extending its biological half-life, and protecting it against losses and faster molecular degradation. Henceforth, the proper size modification, if adopted, will facilitate the BBB crossings, and allow for its increased accumulation at brain sites in the in vivo conditions [29]. The current encapsulate sizes show-cased the possibility and confirmed the preparative and therapeutic aspects of the preparation. 

Interestingly, Patil et al. demonstrated that the half-life of TMZ encapsulated in polymer was extended from 5 to 7 h, as compared with the 1.8 h biological half-life of the pure, free TMZ. They also disclosed that the TMZ nano-preparations were transported to the brain tumor cells, thereby overcoming the resistance to reach the brain sites [26]. Huang et al. [30] synthesized the TMZ solid lipid nanoparticles (TMZ-SLNs), which displayed sustained release. They also discovered that by penetrating the BBB, the TMZ-SLNs efficiently targeted the brain. Also, according to Jain et al. [31], TMZ loaded into PLGA nanoparticles also exhibited prolonged release of the drug with greater cellular uptake. TMZ, when taken orally, was unable to reach the brain’s tumor site, but it was dispersed throughout the body. The TMZ is highly toxic, and the therapeutic doses are limited owing to its significant adverse effects. To prevent the adverse effects, targeted delivery of the TMZ to tumor cells has been considered critical [32]. The tumor cells also increase the drug efflux pumps on the cellular membrane, and lower the drug concentration inside the cell. By encapsulating the drug in distinct nano-carrier, the efflux pumps can be circumvented, and the intracellular uptake of the drug is poised to enhance [33]. Due to the encapsulation feasibility of the PLGA, and the stabilizing effects of the PVA, the TMZ nano-encapsulation was adopted. The PVA also imparted homogeneously layered coating on the TMZ-PLGA-NPs’ surface, and thus provided a stable, non-aggregated nano-formulation [34]. The encapsulation efficiency of the PLGA as TMZ-PLGA-NPs was 79.24%, and the drug loading contents was found to be at 1.13%, although a bit lower drug contents. The bioactivity performance of the prepared TMZ-PLGA-NPs nano-formulation was comparable to similar models of polymer-encapsulated drugs, as described in the literature [35]. 

### 2.2. UV-Vis Spectral Analyses

**Figure 3:**
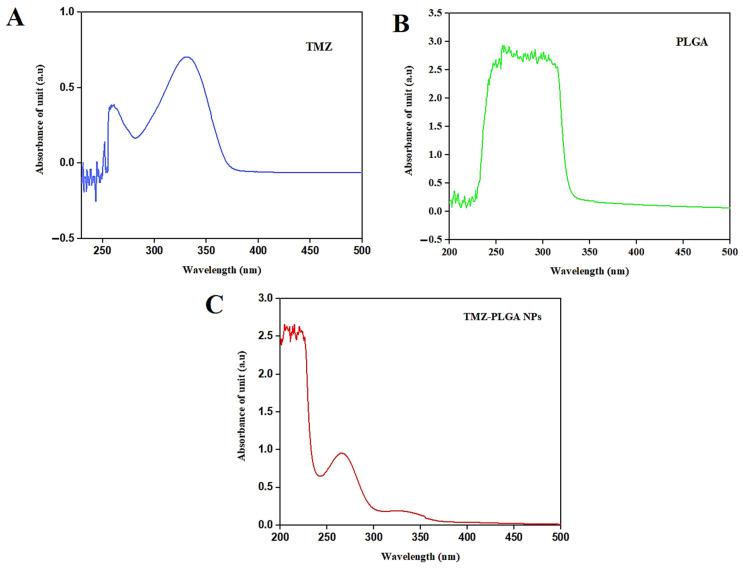
UV-Vis spectra: (**A**) pure, free TMZ; (**B**) PLGA-NPs; (**C**) TMZ-PLGA-NPs.

UV-Visible spectrophotometric measurements were used to confirm the presence of TMZ within the PLGA-NP encapsulates. Figure 3 demonstrated the absorption spectra of the TMZ-PLGA-NPs at a λ_max_ 252 nm, while the pure, free TMZ solution absorption maxima was found to be at λ_max_ 328 nm, which are also reported in a previous investigation [36]. The absorption wavelength value at the λ_max_ absorption of the TMZ-PLGA-NPs was significantly moved from the unloaded, free and pure TMZ, and the PLGA λ_max_ at the 265 nm. This indicated that the drug molecules were safely within the PLGA matrix.

### 2.3. Fourier Transform Infrared Spectroscopic (FT-IR) Analyses

**Figure 4:**
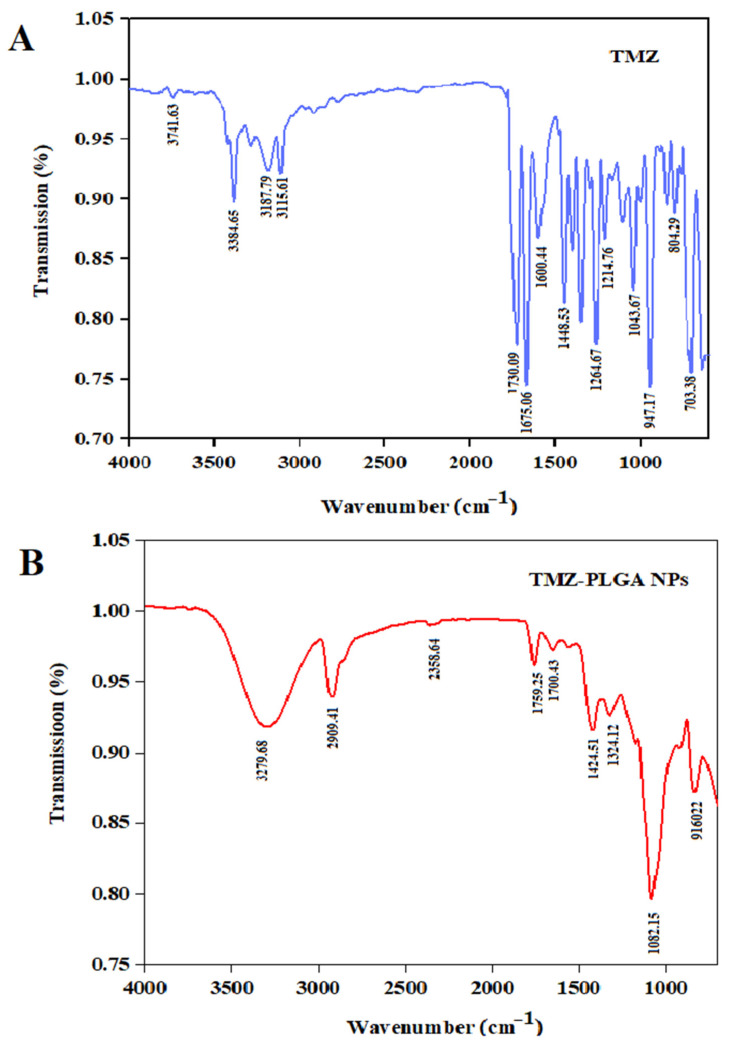
FT-IR spectra: (**A**) pure, free TMZ in blue color; (**B**) TMZ-PLGA-NPs in red color.

FT-IR is among the most efficient approaches used for the estimation of the chemical stability of various compounds following their encapsulation within the nanoparticles [37]. In addition, the FT-IR is a necessary option for determining the functional groups present in drugs and the polymers. As shown in Figure 4, the FT-IR spectra of TMZ and TMZ-PLGA-NPs were observed. The spectra that belonged to the TMZ showed absorption bands at 3384.65 cm^−1^ due to the presence of NH stretching (2^0^ amine, amide). The band at 3115.61 cm^−1^ was due to the asymmetric stretching of alkyl groups. The amide (-NH-C=O) was responsible for the absorption band at 1730.09 cm^−1^. Due to the NH bending, an absorption band at 1675.06 cm^−1^ was observed. The absorption band at 1448.53 cm^−1^ was observed due to the CH bending absorptions. The FT-IR spectrum of TMZ was similar to those reported previously [38].

The IR results also showed an absorption band at 1424.51 cm^−1^ owing to OH and CH bending of the glycolic acid of PLGA, a band at 1324.12 cm^−1^ owing to the C-H bending of the alkyl, and a band at 1082.15 cm^−1^ owing to the O=C–O stretching (from the ester group) for the TMZ-PLGA-NPs. The presence of these distinctive peaks demonstrated the preparation of the PLGA-NPs and without any chemical bondings with the TMZ. The results agreed with those reported for the PLGA encapsulation reported by Arasoglu et al. [39].

### 2.4. X-ray Diffraction Characterizations

**Figure 5:**
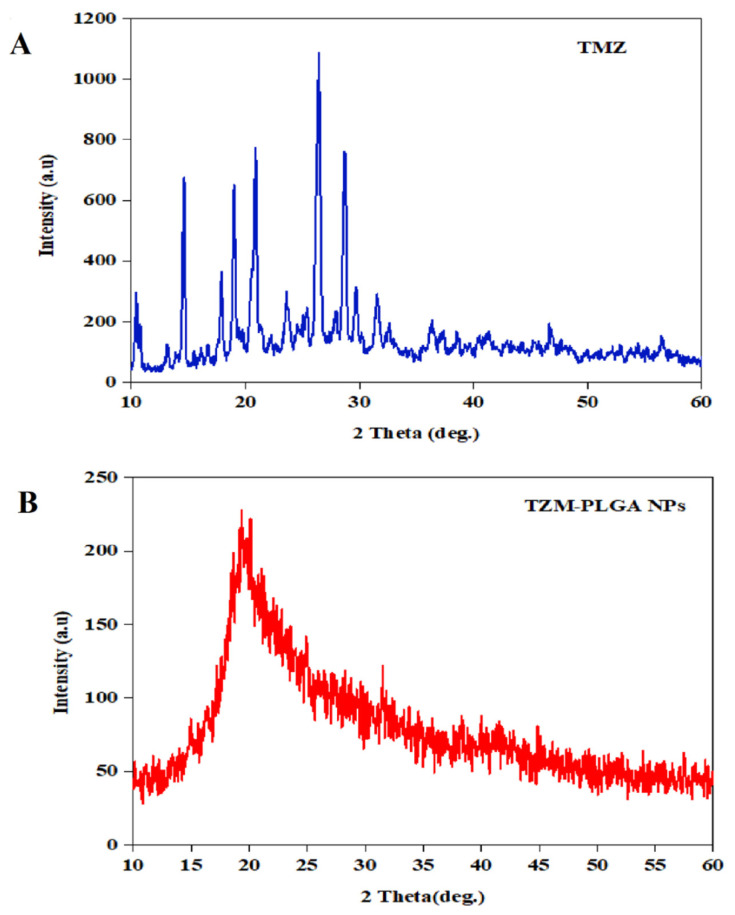
The XRD patterns: (**A**) free, pure TMZ in blue color; (**B**) TMZ-PLGA-NPs in red color.

The XRD (X-ray diffraction), a rapid method for material analysis, was used for measurements of atomic spacing, and provided accurate quantitative information on the atomic arrangements at interfaces of the used materials. The information was useful for identifying the crystal phases present in the sample’s unit cell dimensions, and denoted the physical forms of the crystallinity and amorphousness of the materials [40]. Figure 5 illustrated the XRD patterns of the free and pure TMZ, and TMZ-PLGA-NPs. The free, and pure TMZ showed the entire set of the main sharp characteristic peaks at 2ϴ of 10.4°, 14.45°, 17.15°, 19.85°, 26.6°, and 29.3°, indicating its high crystallinity as concluded for the sharp, narrow and high-intensity diffraction peaks. The XRD peak patterns detected in the TMZ-loaded PLGA nanoparticles, TMZ-PLGA-NPs, showed diffused peaks, therefore indicating the nanoparticles’ non-crystalline nature. These results showed that the TMZ-PLGA-NPs had TMZ molecular dispersions, and the drug was in an amorphous state within the PLGA matrix. The TMZ characteristic peaks’ widening of the TMZ-PLGA-NPs also indicated the drug’s encapsulation within the PLGA matrices [29,41].

### 2.5. Field Emission Scanning Electron Microscopy (FE-SEM)

**Figure 6:**
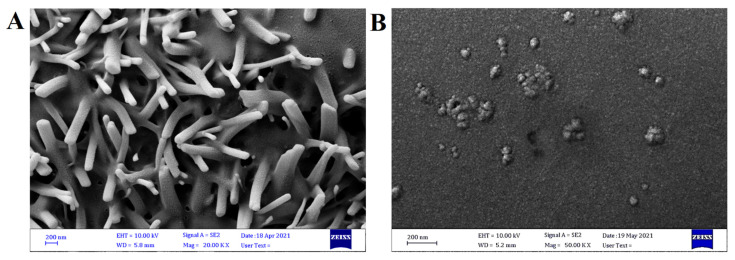
FE-SEM images: (**A**) pure, free TMZ; (**B**) TMZ-PLGA-NPs.

The FE-SEM (Figure 6) was conducted to investigate the sizes, and the morphological features of the pure and free TMZ, and the TMZ-PLGA-NPs. The pure TMZ powder exhibited particles lacking uniformity in size, and appeared to be clumped together in an irregular arrangement. The TMZ-PLGA-NPs showed spherical shapes, with a uniform distribution of sizes. It also showed smooth, and nearly homogenous surface. The particles were observed with a mean diameter of 73.72 nm.

### 2.6. Transmission Electron Microscopy (TEM)

**Figure 7:**
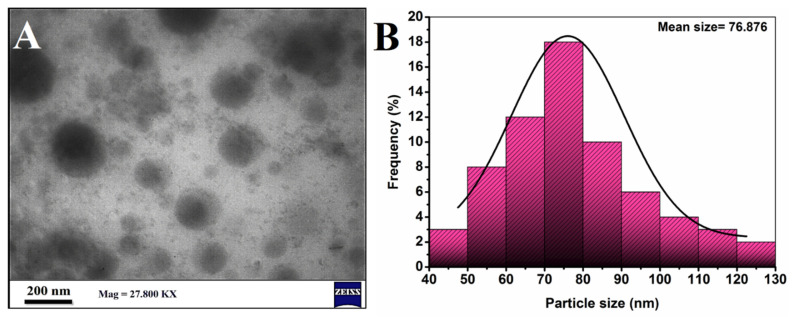
TEM images: (**A**) TMZ-PLGA-NPs; (**B**) particles size distributions.

As seen in Figure 7A, the TEM was used to examine the morphology of the TMZ-PLGA-NPs with scale bar of 200 nm. The images revealed that the TMZ-PLGA-NPs had spherical shapes, with a mean diameter of 76.87 nm. The particles were well separated, indicating that they were stable (i.e., no aggregations). The characteristics of the nanoparticles was determined by a variety of factors, including size, surface charge, and composition [42]. However, nanoparticles size is an important indicator influencing the cellular intake, mechanistics of the biochemical operations, and interactions with the cellular organelles, thereby affecting the cellular mortality, and the systemic clearance. In fact, when it comes to nano-system’s utilization in cancer therapy, the nanoparticles need to be between 10 and 200 nm in sizes to ensure their accumulation within the tumors by virtue of their size, suitable permeability, and retention characteristics [43].

### 2.7. Zeta Potential Analysis

**Figure 8:**
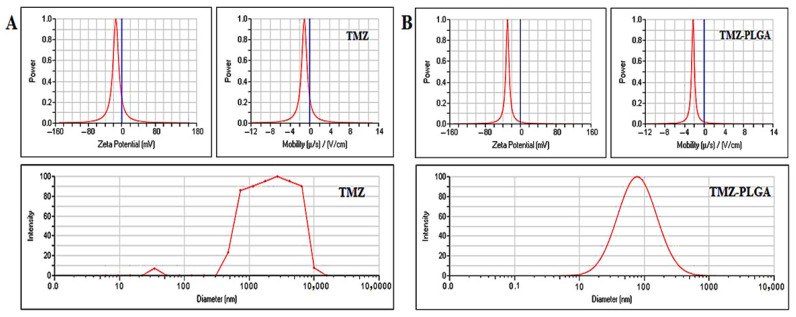
Zeta potential, and DLS analyses: (**A**) pure and free TMZ; (**B**) TMZ-PLGA-NPs.

To further characterize the prepared nano-formulation, the zeta potential of both the pure and free TMZ, and TMZ-PLGA-NPs were assessed (Figure 8A, and B, respectively). The zeta potential of TMZ was −14.34 mV, and the mobility was −1.12 V/cm, while for the TMZ-PLGA-NPs, the zeta potential was −29.25 mV, and the mobility was −2.29 V/cm. The TMZ-PLGA-NPs showed a higher negative zeta potential value, as compared to those of the TMZ alone, thereby suggesting a higher density of negative surface charge resulting from the higher numbers of carboxyl groups [44,45]. Our results revealed a similar observation as those stated by Saraiva et al., who found out that the majority of the published NP formulations for brain delivery exhibited moderate to high negative zeta potentials within values of −1 to −45 mV [46]. With the prepared nanoparticles negative charges being maintained at the higher end of the negative readings, the stability of the particles was confirmed, thus the higher zeta potential value determined the stability of the prepared nanomaterial [47,48]. Reportedly, the high negative, or positive zeta potential values indicate the presence of repulsive forces among the nanoparticles which is sufficient to counteract gravitational attraction, prevent agglomeration, and promote long-term stability [49]. The effectiveness of the NPs systemic circulation, and their uptake by the target cells are influenced by the NPs dimensions, coating, and zeta potential value [50]. Generally, values higher than 30 mV reflect good stability, whereas those higher than 60 mV reflect excellent stability [51]. In addition, a zeta potential value of 20 mV indicates short-term stability, while values in the range of −5 and +5 mV indicate rapid aggregations. Thus, the measures, such as, the desorption rate of the drug in the polymeric NPs, and its loading efficiency are determined by certain primary factors that include the surface charge of the NPs, and the nature of drug’s binding to the polymeric material. In addition, the results of the zeta potential has also been utilized as an indicator of surface attachment or the encapsulation of the drug within the polymer matrix [52]. 

### 2.8. Particle Size Analysis

The Figure 8A, and B (lower lanes, respectively), illustrated the outcomes of the DLS analyses for the pure and free TMZ, and the prepared TMZ-PLGA-NPs. The values of the particle size (nm scale), and the polydispersity index (PDI) were determined. The pure, free TMZ showed particles of approximately 3434 nm mean diameter in size. For the TMZ-PLGA-NPs, the mean particle size was 77.61 nm, whereas the PDI value stood at 0.31 [49]. The PDI values between 0.03 and 0.06 have been considered for monodispersed condition, while the PDI values between 0.1 and 0.2 are known for narrowly distributed particles, whereas the PDI values between 0.25 and 0.5 are undertaken for the broadly distributed particles [53]. Based on standard recommendations, the polymeric nanoparticles need to exert a PDI value below 0.3. The findings of the current investigation revealed that the PDI values of the prepared TMZ-PLGA-NPs were in the acceptable range. Additionally, after the adsorption of an active substance into the terminal groups of the PLGA polymer, an increase in the values of both the particle size, and PDI were observed, and whereupon the zeta potential values were also reduced. One plausible reason for these observations were the presence of the carboxylic end groups of the PLGA molecules on the NPs surfaces [54]. However, only a slight increase was observed in the particles sizes, as compared to the values indicated by the TEM measurements, a difference that was ascribed to the dehydration of the NPs when subjected to TEM analysis. Moreover, the changes in the velocity of the NPs at the time of the DLS measurements were also among the factors that may have affected the variations in the observed particles sizes. In general, when an NP interacts with the dispersion media upon size measurement with the DLS technique, its surface is altered, leading to a reduction in the velocity. Particles tested with DLS for size measurements experience reductions in their sizes, which results in apparently larger particle sizes [55,56]. The development of a localized, nano-sized carrier for drug delivery, capable of crossing the BBB, and reaching the tumor site, the NPs with a size range of 10–200 nm are considered suitable. NPs with a size lower than 10 nm usually experience removal by the renal systems, whereas those with a size of 300 nm, and higher are subjected to elimination through the reticuloendothelial system (RES) [54].

One of the classical approaches to increase the capability of drugs to cross the BBB is to modify the molecular structures of the drugs. For example, Sánchez-López and colleagues prepared a memantine–PEG–PLGA-NPs of particles sizes below the 200 nm, which were able to cross the BBB, both in vitro and in vivo situations, with no cytotoxicity reported on the mouse brain’s microvascular endothelial cells (bEnd.3), and the astrocytes of the rat brain cortex [57]. In another study, the TMZ containing PEGylated NPs, below the 200 nm sizes, were used to deliver combination therapies across the BBB, wherein two intracranial orthotopic mouse models of GBM were utilized [58]. Nanoparticles made of lactoferrin (LF) protein have also been shown to enhance the pharmacological outcomes of drugs, and have shown the specific ability to cross the BBB, as well as target overexpressed Lf receptors on the glioma for enhanced TMZ delivery [59].

### 2.9. In Vitro Drug Release

**Figure 9:**
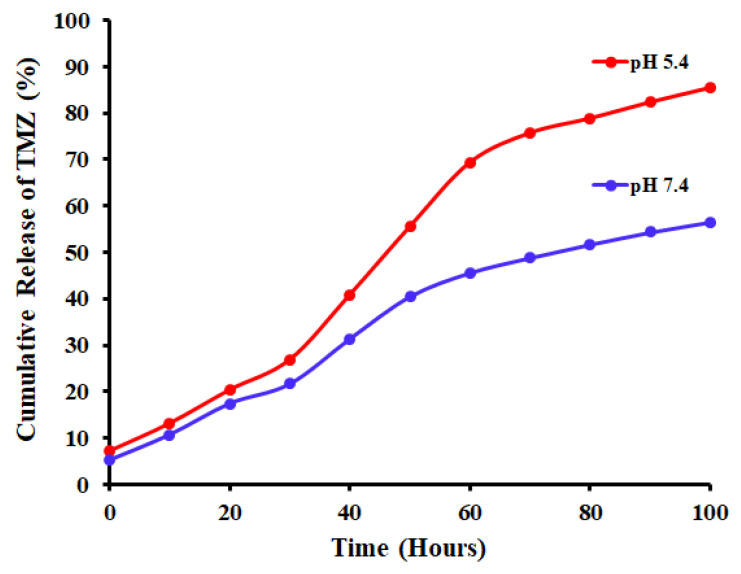
Release profile of TMZ, and modified TMZ-PLGA-NPs in the PBS at pH 7.4 and pH 5.4.

The kinetics of TMZ drug release in both physiological (pH = 7.4) and acidic (pH = 5.0) conditions were analyzed (Figure 9). The UV-Vis spectrometric results demonstrated a pattern of release that depended on the pH of the medium. After 100 h post-treatment, a release of ~85% of the TMZ was recorded at a pH of 5.4, as opposed to only ~56% drug release at a pH 7.4. Earlier investigations also indicated a slow pattern of polymer degradation which enables the drug release [60]. The PLGA-NPs are polymeric nanoparticles that comprise poly lactic acid (PLA) and poly-glycolic acid (PGA), the ratio of which causes changes in the hydrolytic nature of the polymer. Nevertheless, the acidic condition of pH showed higher drug release value for the TMZ from the TMZ-PLGA-NPs in comparison to the release status in basic pH condition. It is likely that these features have contributed to drug release in the tumor, which normally is acidic in nature [61]. 

However, the prepared nanoparticles demonstrated a slow release pattern of the drug when loaded into the PLGA matrix. The half-life of the TMZ was extended as indicated from the dissolution profile. An immediate release of a 30% fraction of the TMZ in the initial 25 h was observed, followed by sustained release of the drug for 100 h, which indicating a biphasic release pattern for the drug, the typical release pattern for PLGA nanoparticles. The developed nanoparticles sustained the drug release, and extended the half-life of the drug. Similar observation was also recorded by Jain et al. [43]. These findings warranted the avoidance of repeated administration of TMZ, and was considered to be of clinical significance. The observation was further confirmed independently, and correlated with the currently observed in vitro evaluation of the drug release against the tested cell line. The in vitro biodegradation-led hydrolysis of the PLGA showed that both, the slight alkaline and strongly acidic media, accelerated the polymer degradation [62]. The nanoparticles underwent rapid and spontaneous changes in their composition and structure, which led to the physical degradation of the TMZ loaded PLGA matrix in a short period of time owing to the breakage of the ester linkages of the PLGA polymeric backbone by pH-conditions driven catalysis. The polymeric erosion was also catalyzed by the protonation of the carboxyl groups present in the PLGA at low pH [63]. 

### 2.10. Cytotoxicity Testings against the AMGM5 and REF Cell Lines

**Figure 10:**
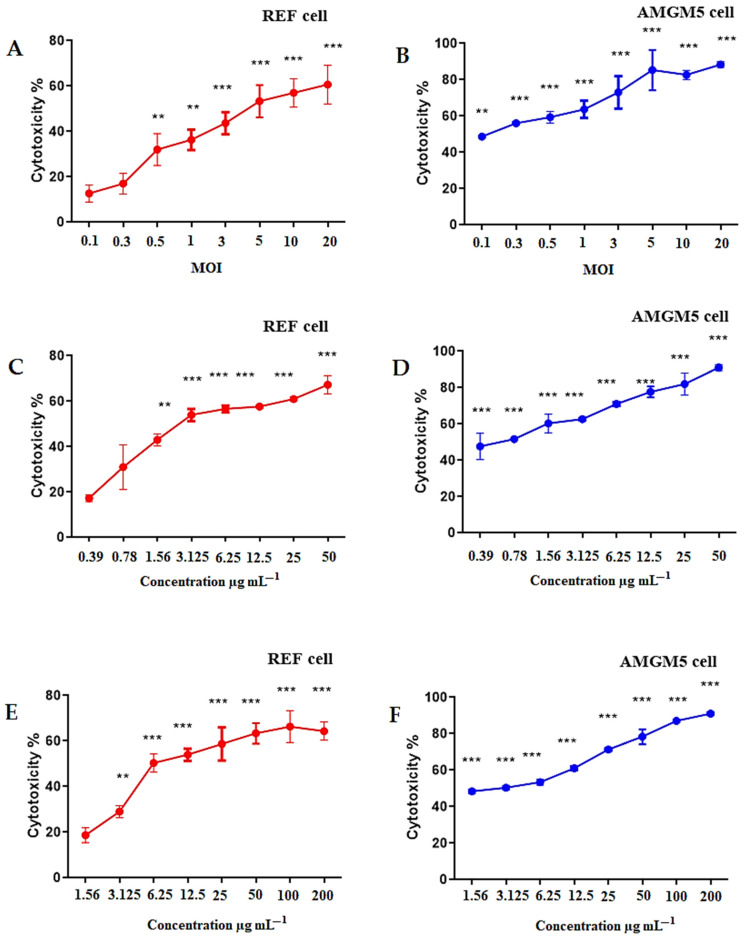
Oncolytic AMHA1 NDV, TMZ, and TMZ-PLGA-NPs cytotoxicity against AMGM5 cancer cells, with low cytotoxicity towards normal embryonic REF cells. The cells were treated with (**A**,**C**,**E**) NDV, TMZ, and TMZ-PLGA-NPs, respectively, on REF cells, and (**B**,**D**,**F**) NDV, TMZ, and TMZ-PLGA-NPs, respectively, on the AMGM5 cell lines for 72 h. Cytotoxicity was investigated using MTT assays. Significance was compared with the control at ** *p* ≤ 0.01 and *** *p* ≤ 0.001.

Figure 10 showed the outcomes of cytotoxicity as tested in MTT assays of the AMGM5 and the REF cells, after 72 h of treatment with various MOI values of the oncolytic AMHA1 NDV, TMZ, and TMZ-PLGA-NPs. The effect of the oncolytic AMHA1 NDV on the growth of the REF cell lines was reflected by a progressive decline (*p* ≤ 0.05) in cell growth, with a decrease in the MOI. The results showed a growth inhibition of 60% at an MOI of 20, and 10% at an MOI of 0.1 (Figure 10A). The TMZ at 50 µg mL^−1^ revealed a significant growth inhibition of 68% in comparison with the 16% growth inhibition caused by 0.39 µg mL^−1^ dose (Figure 10C). The TMZ-PLGA-NPs at a concentration of 200 µg mL^−1^ exhibited a significant growth inhibition of 65% in comparison to the 19% growth inhibitions caused by the 1.56 µg mL^−1^ dose concentration (Figure 10E).

Figure 10B showed the cytotoxic effects on the AMGM5 cell lines after 72 h of treatment with 20 MOI viral particles, which showed a highly significant growth inhibitions of 88.5%, whereas a lower inhibition rate of 48.5% was observed at 0.1 MOI. These results suggested that the NDV required a greater number of molecules to enter the cells, and interfere with the cell receptors, cytoplasmic organelles, and/or nuclear membrane, thereupon resulting in cancer cells death. The Iraqi oncolytic strain of NDV was demonstrated to have killing activity against tumor cells based on several mechanisms including eliciting an s immune response that specifically attacks infected tumor cells [64]. The Iraqi strain of NDV (AD2141), especially in human cancer cell lines, caused genotoxicity by inducing DNA fragmentation and activating FasL levels [65]. Because of its rational safety profile and associated cell death, which promotes local and distant antitumor immune responses, the Newcastle disease virus is a promising oncolytic agent. It also has several distinct characteristics and is less expensive than the modified recombinant strains, which require extra procedures for viral product genetic control [66,67]. It has strong cell-binding characteristics, specifically on tumor cells, where it multiplies selectively. It is also reasonably safe and has been examined in various cancer cell lines [68]. Therefore, the mechanism of action of NDV consists of two parts, namely, direct cell lysis and cancer unmasking by immunogenic cell death [69]. Intratumoral injection of the Iraqi NDV strain revealed a strong immune-stimulatory impact, as it induced IL-2 and IFN-gamma and attracted CD8 cytotoxic T cells and CD56 natural killer lymphocytes into the infected tumor tissue [70,71]. 

With regard to TMZ, the highest growth inhibition rate was recorded at of 50 µg mL^−1^ (90.5%), while at 0.39 µg mL^−1^, the growth inhibition was 47% (Figure 10D). TMZ-PLGA-NPs at a concentration of 200 µg mL^−1^ were more effective and revealed a higher growth inhibition of 93% compared with a concentration of 1.56 µg mL^−1^, which showed a 47.5% inhibition rate (Figure 10F). However, the results revealed that TMZ-PLGA-NPs were more effective against AMGM5 cells than TMZ alone. Less cytotoxicity observations were recorded with the normal cell line. The IC_50_ values for NDV MOI were 5.71 for REF cells and 1.60 for AMGM5 cells. The IC_50_ values of TMZ were 2.84 μgmL^−1^ for REF cells and 0.78 μgmL^−1^ for AMGM5 cells, while the IC_50_ values of the TMZ-PLGA-NPs were 6.08 μgmL^−1^ for REF cells and 1.68 μgmL^−1^ for AMGM5 cells.

TMZ is an agent that renders DNA more alkaline and elicits cell cycle arrest at G2/M, ultimately resulting in apoptosis [72]. Chemotherapy involves the utilization of alkylating agents that raise DNA damage caused by genotoxic stress [73]. This mechanism is considered the most critical biological response to DNA damage. Following DNA damage, this response is immediately regulated by certain intracellular activities including DNA repair and cell cycle arrest [74,75]. 

In the interval between sample preparation and interaction with the media, free TMZ rapidly hydrolyzes into inactive metabolites. After the placement of TMZ within the PLGA carrier, however, no hydrolysis process takes place, maintaining and extending the cytotoxic action with progress in time. PLGA-dependent nanoparticles cause a remarkable improvement in the stability of TMZ while maintaining the cytotoxic efficacy. These features render them ideal for protecting the TMZ structure against hydrolysis while also increasing its cytotoxicity. According to cell quantification, our results are consistent with what was found in a study by Di Martino et al. [76].

### 2.11. Synergistic Effects of the TMZ, NDV, and TMZ-PLGA-NPs Combinations Utilizing Cytotoxicity Assay and Chou–Talalay Analysis of the AMGM5 Cell Lines

**Figure 11:**
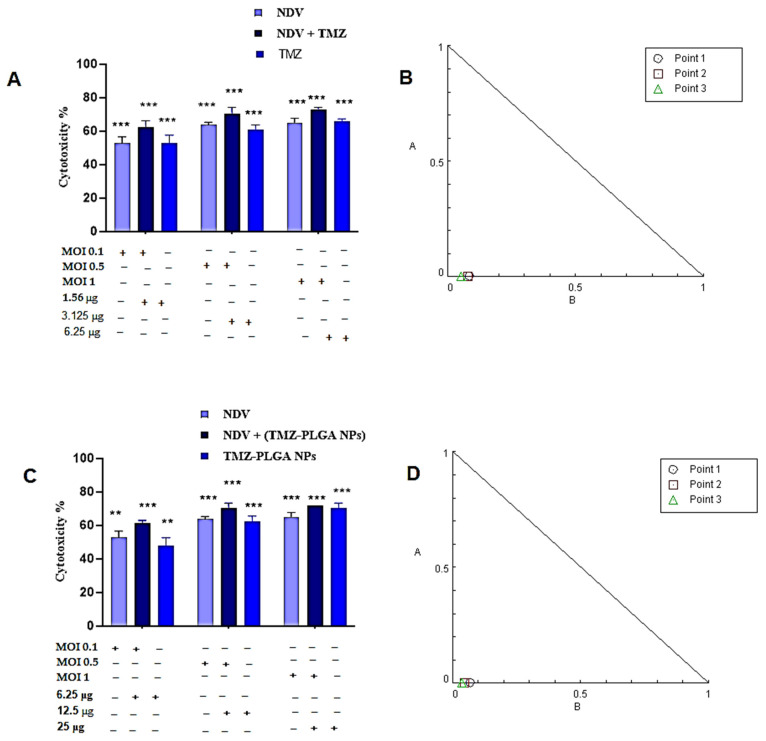
(**A**,**C**) The combinations of NDV and TMZ; NDV and TMZ-PLGA-NPs showed superior anticancer activity on AMGM5 cell lines when treated with IC_50_ concentrations, respectively. The Figure 11 (**B**,**D**) Illustrates the normalized isobolograms of the non-constant combination ratios measured by the Chou–Talalay method, wherein the CI values can be quantitatively defined as the synergism (CI < 0.9), additive effects (CI = 0.9–1.1), and antagonism (CI > 1.1). Data are from three different experiments. Significance was compared to the control at ** *p* ≤ 0.01, and *** *p* ≤ 0.001.

To analyze the impacts of the oncolytic NDV, TMZ, TMZ-PLGA-NPs, NDV and TMZ, as well as NDV together with the TMZ-PLGA-NPs combination therapies, the current study examined the cytotoxicity ratios of the NDV (i.e., 0.1, 0.5, and at 1 MOI), TMZ (i.e., at 1.56, 3.125, and at 6.25 μgmL^−1^), TMZ-PLGA-NPs (i.e., at 6.25, 12.5, and at 25 μgmL^−1^), NDV and TMZ (i.e., at 0.1 + 1.56, 0.5 + 3.125, and at 1 + 6.25 μgmL^−1^), as well as NDV and TMZ-PLGA-NPs together (i.e., at 0.1 + 6.25, 0.5 + 12.5, and at 1 + 25 MOI and μgmL^−1^, respectively) as presented in Figure 11. 

The synergetic effects against the AMGM5 cell lines were noticed upon treatment with all combinations and the doses tested. The CompuSyn^®^ isobologram was applied to extract the CI values based on the dose–effect results of both the single and combined treatments. A CI < 0.9 implies synergism, a CI at 0.9 to 1.1 implies an additive effect, while the value for CI > 1.1 implies the antagonism. The AMGM5 cell lines showed the CI values for all the combinations reflecting synergism, or interactions between the NDV and TMZ (Figure 11A,B), and between the NDV and TMZ-PLGA-NPs (Figure 11C,D). In addition, the combination treatments showed higher growth inhibition rates than that observed for the NDV, and the TMZ alone (Table 1A,B).

The findings of the cytotoxicity experiments on cancer cells (AMGM5) revealed the synergistic impacts between the virulent NDV strain and the drug TMZ, as well as between the NDV and the TMZ-PLGA NPs, at the lowest and highest dosages. In addition, the findings of this experiment demonstrated that combining the NDV and TMZ-PLGA-NPs caused higher growth inhibition rate in comparison to the either the pure and free TMZ, or the TMZ-PLGA-NPs alone as monotherapy. The combination therapies showed the advantages of improved efficacy, and reduced drug resistance. As a result of these benefits, the strategy is expected to become a powerful method in the development of treatments for other cancers also [77]. Thus, the goal of NDV combination therapy was to employ the designated medication that lessens the probability of cancer cells developing resistance [78].

However, despite extensive glioblastoma treatment strategies available, which includes resection, radiotherapy, and chemotherapy, the majority of GBM patients have experienced regression. In a subset of cancer patients with the methylated MGMT gene, the therapy with alkylating drugs, such as TMZ, have been found useful; nonetheless, the patients who have an unfavorable MGMT condition, have showed resistance to TMZ medication [79,80]. Furthermore, most cancers are known to develop resistance to chemotherapy during treatment, and this is associated with switching from methylated to an undesired non-methylated MGMT state. The treatment with TMZ, according to Felsberg and colleagues, improved the survival of tumor cells with non-methylated MGMT status, and thus leading to resistant clones that result in cancer regression [81]. As an expected consequence of the situation, the current study focused on re-establishing the chemotherapeutic agent’s sensitivity in MGMT-expressing cells using the combination of NDV and the TMZ treatment. The in vitro therapy of the glioma cells with NDV has shown to be a unique and promising strategy so for towards combating the GBM. Additionally, the NDV combined with chemotherapeutic agents have been the focus of several studies. For example, when paired with other anticancer agents, the NDV has been considered as an anti-hematological malignancy substance with high potential. On plasmacytoma, and non-Hodgkin’s lymphoma cells, the NDV was discovered to be synergistic in effects with the doxorubicin. When coupled with rituximab, another anti-cancer agent, the cytotoxicity of the same type of cells increased [21]. Also, the NDV have been shown to enhance apoptotic responses by inhibiting the AKT signaling, since TMZ and NDV can exert various influences on the AKT signaling [82,83].

### 2.12. Colony Formation Assay

**Figure 12:**
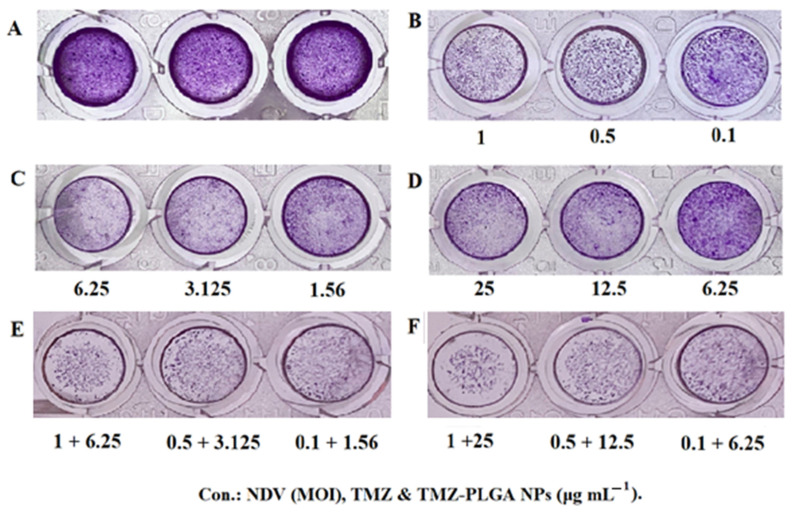
Colony formation assay of the AMGM5 cancer cells: (**A**–**F**): untreated cells, NDV, TMZ, TMZ-PLGA-NPs, NDV and TMZ, NDV and TMZ-PLGA-NPs, respectively.

A colony formation assay for AMGM5 cell lines was performed to assess the long-term toxic impacts of the NDV, TMZ, TMZ-PLGA-NPs, NDV and TMZ, and NDV and TMZ-PLGA-NPs at the three doses used. Figure 12 showed the results of treatments with NDV, TMZ, and TMZ-PLGA-NPs, NDV and TMZ, and NDV and TMZ-PLGA-NPs at the MOI and concentrations as described in Section 3.9. The results demonstrated a concentration-associated reduction in the number of AMGM5 cell colonies as compared with the untreated cells. The treatment with NDV, TMZ, and TMZ-PLGA-NPs, all alone, had a mild inhibitory effect against the AMGM5 cells. The monotherapy was less effective than the combined therapies. The combination therapy of NDV and TMZ, NDV and TMZ-PLGA-NPs induced changes to the cell’s morphology, and that resulted in clusters with fewer extensions, as well as reduced connectivity between the treated cells. However, the untreated cells did not show these changes. Similar observations were reported earlier [31].

### 2.13. Morphology and Quantitative Image Analyses Using Crystal Violet

**Figure 13:**
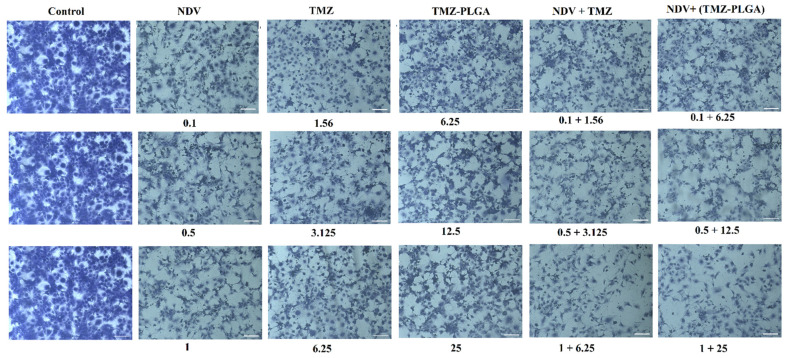
The treated and untreated cells underwent cytomorphology using crystal violet stain. Concentrations: NDV (MOI); TMZ and TMZ-PLGA-NPs (µg mL^−1^). Scale bar = 20 µm.

As shown in the majority of the examined fields (Figure 13), the treated cells, comparatively, had fewer numbers of cells, and induction of morphological alterations, such as, cell shape alterations were observed. Moreover, the cells treatment caused the cells to form clusters and have fewer cellular extensions. Furthermore, the treated cells also had contracted nuclei, while the untreated cells had nuclei with normal morphology and showed normal cellular shapes and sizes. These findings imply apoptosis as a possible pathway of cell death. These effects were clearly increased after combination therapy treatment, and NDV and TMZ-PLGA-NPs were found more potent as compared with the other treatments, both as monotherapy and as the combined therapy. A previous investigation showed similar results by use of TMZ, and TMZ-PLGA-NPs on C6 glioma cells. The untreated cells exhibited abnormal nuclei that showed pleomorphism, which were indications of human C6 glioma cancer. The spindle-shaped cells of C6 glioma were found to experience distortion following the treatment with free TMZ, and TMZ-loaded formulation [31].

### 2.14. Acridine Orange–Propidium Iodide (AO/PI) Dual-Staining Assay

**Figure 14:**
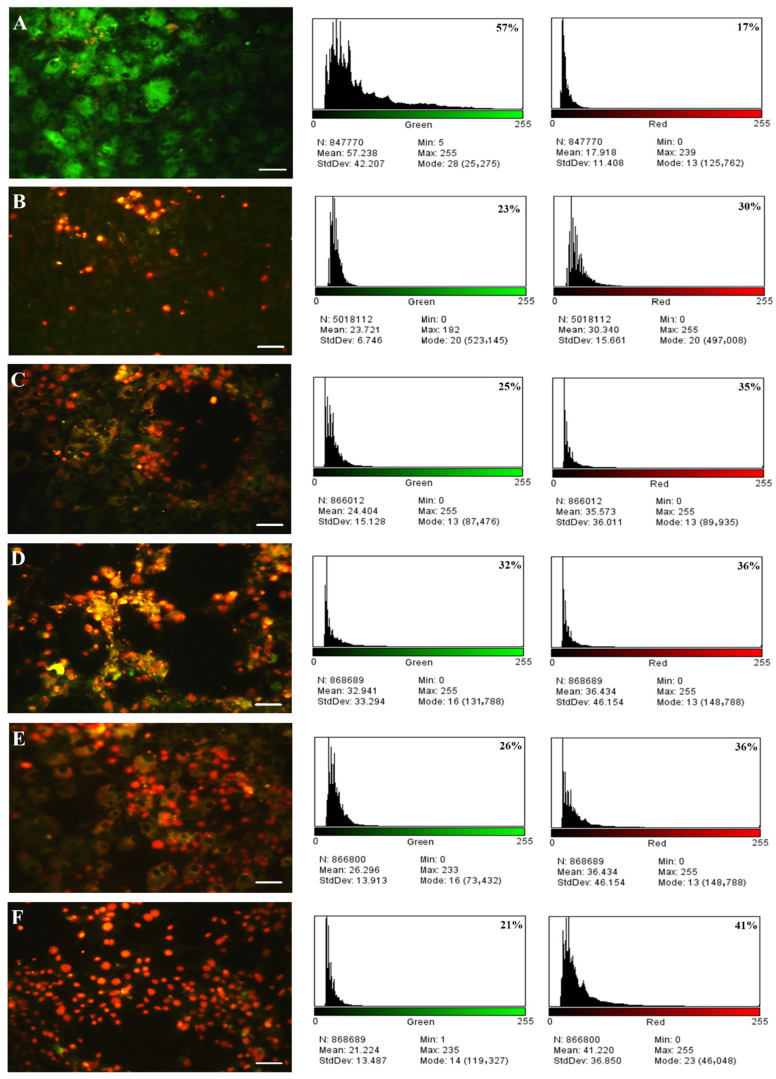
Fluorescent micrograph of acridine orange, and propidium iodide double-stained AMGM5 cells. The histogram images showed the percentage of green fluorescence (viable cells), and red fluorescence (dead cells) for each treatment: (**A**) Untreated cells; (**B**–**F**) NDV, TMZ, TMZ-PLGA-NPs, NDV and TMZ, and NDV and TMZ-PLGA-NPs, respectively. Cells were treated with the IC_50_ concentration. Scale bar = 20 µm.

As shown in Figure 14, a fluorescent microscope with AO-PI double staining was used to validate the observed changes in the shapes of the nuclei of the treated AMGM5 cells after their exposure to the viral particles (0.5 MOI). These viral particles revealed synergism with TMZ at a concentration of 3.125 µgmL^−1^, with TMZ-PLGA-NPs at a concentration of 12.5 µgmL^−1^, with NDV and TMZ at a concentration of MOI 0.5 + 3.125 µgmL^−1^, and with NDV and TMZ-PLGA-NPs at a concentration of MOI 0.5 + 12.5 µgmL^−1^. In addition, the percentage of the dead cells by apoptosis in the treated AMGM5 cells were calculated and displayed (Figure 14).

**Figure 15:**
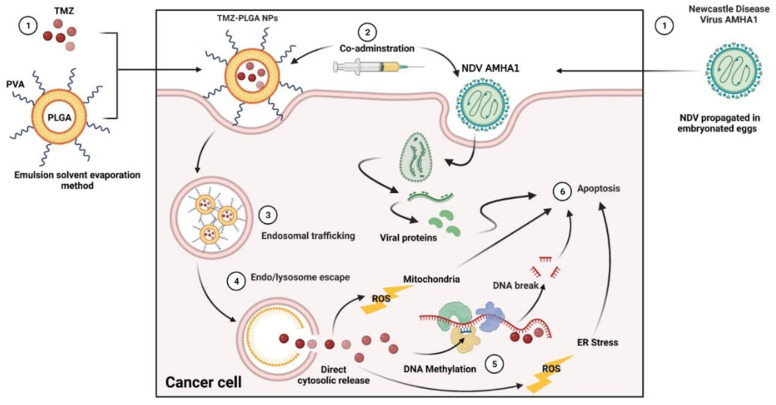
The proposed mechanism of NDV + TMZ-PLGA-NPs against cancer cells.

The untreated cells exhibited nuclei with an unaffected shape, high stability, and bright green color. However, morphological alterations, examined with fluorescent agents revealed significantly increased changes, including chromatin condensation in the treated cells. Upon apoptosis, the cells exhibited nuclei with colors that varied between red and orange, whereas the chromatin exerted various levels of condensation or fragmentation based on the color variations. However, the changes noticed in the morphology of the treated cells implied an apoptotic, and not a necrotic pathway. The intensity of the red color increased with NDV and TMZ-PLGA-NPs, which was more potent than the NDV and TMZ combined therapy, indicating the strength of the synergistic impact of the NDV and TMZ-PLGA-NPs on the cell lines. When compared to the untreated cells, the cells demonstrated an aggressive membrane breakdown upon exposure to treatments. All of these alterations in the cells were caused by a shift in their osmotic equilibrium, which resulted in more exhaustion of the cellular molecules outside of the cells. Many theories exist for why the cancer cells die, including interactions with their target proteins that directly generate DNA damage by inhibiting enzymes’ ligase domain while leaving the nuclease regions undamaged, thus allowing the enzyme to break the DNA without ligation again [84]. Thus, the DNA damage might be caused by oncolytic NDV pathways together with the cytotoxic action of the delivered NPs. The combination therapy is predicated on taking risks to achieve the maximum results at the lowest feasible concentrations [85], and these concentrations were used to expose cell lines to synergistic virus particles (MOI). Hence upon, a proposed mechanism of cell death is illustrated (Figure 15). 

However, he TMZ is capable of inducing the generation of amounts of ROS that are sufficient to kill cells, as reported by Yin et al. [86]. In an earlier investigation that presented TMZ-PLGA-NPs and Cmab-TMZ-PLGA-NPs, induction of ROS generation was observed in the U-87 MG cell lines. Moreover, the Cmab-TMZ-PLGA-NPs elicited higher levels of ROS compared with the other two treatments. They also resulted in a high accumulation of TMZ in U-87 MG cells, leading to DNA methylation and its subsequent fragmentation [87,88]. Upon exposure of tumor cells to DNA damage, as a result of treatment with chemotherapeutic agents, such as TMZ, double-stranded breaks (DSBs) were formed, which readily resulted in phosphorylation of histone H2A variant H2AX. The phosphorylated version of H2AX is termed γH2AX [89]. As the generation of γ-H2AX occurs during DNA damage, it is supposed to lead to the provisions of means to monitor the efficacy of the anticancer treatments. It also helps to evaluate how sensitive the tumor cells are to the chemotherapeutic agents that functioned through DNA damage [90].

## 3. Materials and Methods

### 3.1. Materials and Reagents

Temozolomide (TMZ) was bought from Santa Cruz Biotechnology; polylactic-co-glycolic acid (PLGA) (50:50, Mw 24,000–38,000) and polyvinyl alcohol (PVA; 98% pure) were supplied by Sigma Chemical Co. (St. Louis, MO, USA). The Iraqi isolate (ICCMGR/Najaf/2013) of the Newcastle disease virus was provided by the Department of Experimental Therapy, Iraqi Center for Cancer and Medical Genetic Research (ICCMGR), Mustansiriyah University, Baghdad, Iraq. The viral seeds were stored at −86 °C in a deep freezer. Fertilized eggs were obtained from the Al-Khalil Hatchery, Baghdad, Iraq. Chicken red blood cells were collected from the brachial vein under the right wing of the chicken. The dyes used in the present study (i.e., acridine orange, ethidium bromide and crystal violet) were provided by Sigma Chemical Co. (St. Louis, MO, USA). 3-(4,5-Dimethylthiazal-z-yl)-2,5-diphenyltetrazolium (MTT) (Elab Science Biotechnology Inc., USA), trypsin-EDTA, dimethyl sulfoxide (DMSO), Roswell Park Memorial Institute (RPMI)-1640, minimum essential medium (MEM), fetal calf serum (FCS), and antibiotics (i.e., ampicillin: 0.5 g; streptomycin: 1 g) were ordered from Biosource International, Nivelles, Belgium.

### 3.2. Cell Line Cultures

The human glioblastoma cancer cell line (Ahmed-Majeed-Glioblastoma-Multiforme-2005, AMGM5), the normal cell line (rat embryo fibroblasts (REFs)), and the Vero cell line were produced from a healthy adult African green monkey’s kidney. Cell lines were obtained from the ICCMGR. Cells were cultured in Falcon T 25 cm^2^ flasks containing RPMI 1640 medium supplemented with 10% FBS, 2 mM L-glutamine, and 20 mM HEPES in 5% CO_2_ at 37 °C. Similar conditions were applied for culturing Vero cells, except the culture media were replaced by MEM.

### 3.3. Preparation of the TMZ-Loaded PLGA Nanoparticles

**Figure 16:**
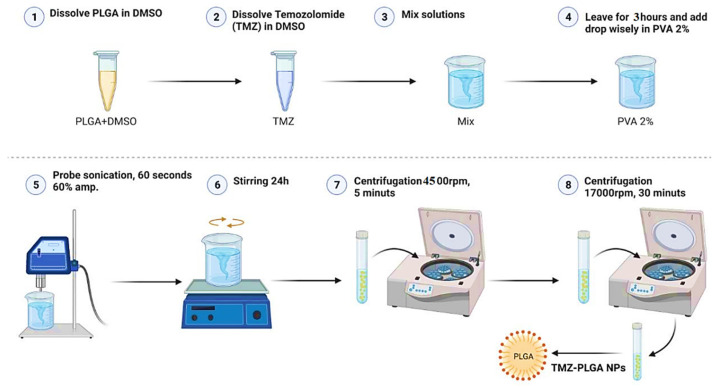
Preparation steps for the TMZ-PLGA-NPs using the emulsion solvent evaporation method.

With some modifications to the nanoprecipitation method [91], this technique was used to prepare TMZ-loaded PLGA nanoparticles [91]. The method is schematically presented in Figure 16. To obtain a homogeneous solution, 5 mg of TMZ was dissolved in 1 mL of dimethyl sulfoxide (DMSO) and sonicated for 10 min. Afterward, 1 mL of DMSO was used to dissolve 20 mg of PLGA, followed by mixing with TMZ solution and leaving it for 3 h with the dropwise addition of 2% aq. PVA solution. 

To maintain the active component in place, a PVA solution (2%; 4 mL) was used as a stabilizer followed by the dropwise addition of PLGA, and homogenization was performed by stirring, before sonicating with a probe sonicator (60 s; 60% amp.). The emulsified solution was then mixed with 20 mL of deionized, distilled water to assist in diffusion, followed by overnight agitation at 25 °C to ensure complete encapsulation. The resultant particles’ suspension was rotated at 4500 rpm for 5 min. The supernatant was separated for ultracentrifugation at 17,000 rpm for 25–30 min to isolate the free active compounds and the unbound stabilizing agents from the particles in the solution. Following the separation of the supernatant, the obtained pellet was dried and dispersed in 20 mL deionized, distilled water, followed by its evaporation at room temperature. The percent of encapsulated TMZ by the nanoparticles was calculated using the following equations:

(1) $$\mathrm{Encapsulation}\;\%=\left(\mathrm{Weight}\;\mathrm{of}\;\mathrm{TMZ}\;\mathrm{in}\;\mathrm{NPs}/\mathrm{Weight}\;\mathrm{of}\;\mathrm{initial}\;\mathrm{drug}\right)\times 100$$

(2) $$\mathrm{Drug}\;\mathrm{Loading}\;\%=\left(\mathrm{Weight}\;\mathrm{of}\;\mathrm{TMZ}\;\mathrm{in}\;\mathrm{NPs}/\mathrm{Weight}\;\mathrm{of}\;\mathrm{NP}\right)\times 100$$

### 3.4. Characterization of the Temozolomide Nanoparticles

Initial examinations of the TMZ, PLGA, and TMZ-PLGA-NPs were achieved by utilizing UV–Vis spectral measurements (Shimadzu Europe-UV-1650PC Spectrophotometer, Tokyo, Japan) with continuous scanning at 200–900 nm. An FT-IR spectrophotometric examination was performed on an FTIR 8400S instrument (Tensor 27, Netherlands) with a spectral range of 4000–400 cm^−1^ and a resolution of 4 cm^−1^. The machine was operated in the attenuated total reflection mode. The prepared NPs were analyzed using an X-ray diffractometer (XRD-6000, ADX-2700, Stoughton, MA, USA) at a current of 30 mA and a voltage of 40 kV. The NPs’ diffraction patterns were characterized under a Cu Kα incident beam (λ = 1.542 A^o^) at 2θ = 5–40°, whereas a Brookhaven Zeta PALS machine (Milton Keynes, UK) was employed to assess the zeta potential and investigate the particle size. A field emission scanning electron microscope (FESEM) (MIRA 3 TESCAN, Brno, Czech Republic) was employed to morphologically characterize the prepared formulation. The morphology as well as the distribution of the preparations were also examined through TEM visualization (Zeiss, Germany) at a voltage of 400 kV.

### 3.5. In Vitro Release of TMZ

The TMZ was released from the PLGA-NPs, and for this purpose, TMZ-PLGA-NPs were incubated in buffer solutions that mimicked the blood pH (7.4) and that of the endosomes of the cancer cells (5.4) based on the procedure reported by Zhang et al. [29]. In brief, a dispersion solution of 5 mg mL^−1^ of TMZ-PLGA-NPs and 5 mL of PBS (pH 7.4 and 5.4) were prepared, followed by shaking–incubation (at 37 °C) with stirring (100 rpm). Then, 1 mL of PBS from the vial was withdrawn after incubation for 0, 1, 5, 10, and 20 h and up to 100 h. UV-Vis spectra (328 nm) were measured to obtain the absorbance of the solutions from all of the treatments. The concentration of the released TMZ was determined by applying the following equation together with the use of a standardized calibration curve:

(3) $$\;\mathrm{Cumulative}\;\mathrm{Release}\;\mathrm{of}\;\mathrm{TMZ}\;\%=\left(\mathrm{Wr}/\mathrm{Wt}\right)\times 100$$

where Wr and Wt represent the actual and theoretical TMZ weight values, respectively, in each release.

### 3.6. NDV Propagation

The Department of Experimental Therapy, ICCMGR, donated the NDV (Iraqi AMHA1 strain). An allantoic fluid extract of embryonated chicken eggs, provided by Al-Kindi Company, Baghdad, Iraq, was employed in the production of stock attenuated NDV. Cleaning the stock from the debris was achieved through centrifugation at 3000 rpm at 4 °C for 30 min. NDV was measured using the hemagglutination test and then liquated and kept at a temperature of − 80 °C. Viral titers were measured using a classical approach that included the titration of an infective dose of 50% on Vero cells [92].

### 3.7. Cytotoxicity against the REF Normal Cell Line and the AMGM5 Cancer Cell Line

Cells (1 × 10^4^ cells mL^−1^) were first subjected to seeding in a 96-well microplate and incubated at 37 °C for 24 h. The formation of monolayer confluence was visualized using an inverted microscope. The 3-(4,5-dimethylthiazol-2-yl)-2,5-diphenyltetrazolium bromide (MTT) assay was performed for investigating the possible cytotoxic impacts of the tested preparations [93]. To determine the IC_50_ values, the cells were treated with various concentrations of TMZ (i.e., 0.39, 0.78, 1.56, 3.125, 6.25, 12.5, 25, and 50 μgmL^−^^1^) and TMZ-PLGA-NPs (i.e., 1.56, 3.125, 6.25, 12.5, 25, 50, 100, and 200 μgmL^−^^1^), along with various MOI (multiplicities of infection) values of NDV (i.e., 0.1, 0.3, 0.5, 1, 3, 5, 10, and 20). After 72 h, the wells were treated with an MTT dye solution (50 μL, 2 mgmL^−1^), followed by incubation (3 h) and the addition of 150 μL DMSO (100%) to solubilize the formazan crystals. The plates were incubated for 15 min at 37 °C to remove air bubbles. An ELISA plate reader (ELx 800, Bio-Tek Instruments Inc., Winooski, VT, USA) operated at 570 nm was utilized to measure the optical density (OD) of the treated and untreated cells. The rates of cell growth inhibition (cytotoxicity %) were determined using the following equation:

(4) $$\mathrm{Cytotoxicity}\;\%=\left(\mathrm{OD}\;\mathrm{control}-\;\mathrm{OD}\;\mathrm{sample}/\mathrm{OD}\;\mathrm{control}\right)\times 100$$

where OD control and OD sample represent the optical density values of the untreated and treated cells, respectively [94].

### 3.8. Combined Cytotoxicity Assays and Chou–Talalay Analysis

The outcomes of the cytotoxicity assays for the IC_50_ determinations were used as the basis for selection of doses. The IC_50_ concentrations for NDV, TMZ, TMZ-PLGA-NPs, NDV and TMZ, and NDV and TMZ-PLGA-NPs were selected against the AMGM5 cell lines. Seeding of the AMGM5 cells (1 × 10^4^ cells mL^−1^) was achieved in 96-well plates, followed by overnight incubation. As an initial step, initially the plates received NDV at MOI values of 0.1, 0.5, and 1. Thereafter, the IC_50_ serial dilution values of TMZ alone (i.e., 1.56, 3.125, and 6.25 μgmL^−1^), TMZ-PLGA-NPs alone (i.e., 6.25, 12.5, and 25 μgmL^−1^), NDV and TMZ (together with the MOI, i.e., 0.1 + 1.56, 0.5 + 3.125, and 1 + 6.25), and NDV and TMZ-PLGA-NPs (0.1 + 6.25, 0.5 + 12.5, and 1 + 25) were added to assess the cell growth inhibition rates [95]. The growth inhibitions were measured after 72 h of infection using the MTT assays. As described earlier, at 72 h post-infection, the above-described MTT test were performed for assessment of the cells growth inhibition rates. All tests were conducted in triplicate, whereas the synergism was determined based on studying the constant ratios of the dosages of each tested treatment. CompuSyn^®^ software (CombuSyn Inc., Paramus, NJ, USA) was applied for the calculation of the Chou–Talalay combination index (CI), aimed at analyzing the effects of the combinations of NDV and TMZ and NDV and TMZ-PLGA-NPs. The CI values were assessed based on the unfixed ratios of NDV and TMZ, and mutually exclusive equations. A CI value of 0.9–1.1 reflects the additive effects, <0.9 reflects synergism, and >1.1 reflects antagonism [96].

### 3.9. Clonogenicity Survival Assay

The slightly modified method of Franken et al. was employed to determine the activities of the tested materials in inhibiting colony formations by the cells [97]. In brief, the AMGM5 cells were seeded in 96-well plates for 24 h, followed by treatment with the tested values of NDV (i.e., 0.1, 0.5, and 1 MOI), TMZ (i.e., 1.25, 3.125, and 6.25 μg mL^−1^), TMZ-PLGA NPs (i.e., 6.25, 12.5, and 25 μg mL^−1^), NDV and TMZ (i.e., 0.1 + 1.56, 0.5 + 3.125, and 1 + 6.25), and NDV and TMZ-PLGA-NPs (i.e., 0.1 + 6.25, 0.5 + 12.5, and 1 + 25). The medium was replaced with PBS at 72 h post-treatment, followed by fixation, staining with crystal violet, removal of excess strain and, lastly, photography.

### 3.10. Morphology Analysis

After seeding of the cells (1 × 10^4^ cells mL^−1^) in the 96-well microplate, and their incubation (37 °C, 24 h), the formation of monolayer confluence was visualized under an inverted microscope. At 72 h post-treatment with the above-described doses of the different tested materials, the medium was replaced by PBS, followed by fixation, staining with crystal violet, and washing to remove the excessive stain. An inverted light microscope supplied with a digital color camera (Leica Microsystems, Germany) was utilized to photograph the treated and untreated cells from the randomly chosen cultures [32].

### 3.11. Assessment of Apoptosis

Propidium iodide/acridine orange was used to investigate cell death by apoptosis of the infected and controlled AMGM5 cells. PI/AO dual-staining tests were performed. The cells (7 × 10^3^ cells mL^−1^) were initially subjected to an overnight seeding in the 96-well plate and treated with IC_50_ concentrations of NDV (MOI 0.5), TMZ (3.125 μgmL^−1^), TMZ-PLGA-NPs (12.5 μgmL^−1^), NDV and TMZ (0.5 + 3.125), and NDV and TMZ-PLGA-NPs (0.5 + 12.5) with incubations at 37 °C for 72 h. The classical PI/AO staining protocol was then applied by adding 50 μL of AO/PI staining mixture to 1 mL of cell suspension. The preparations were then left to stand for 30 s at ambient temperature. After discarding the staining solution, the samples were directly photographed with the use of a Leica fluorescent microscope (Leica Microsystems, Wetzlar, Germany), and Image J software (http://rsb.info.nih.gov/ij/ accessed on 3 April 2022) was used to examine the images. Each photo was quantitatively measured in three different ways for statistical analysis. The percentage (%) of cells stained with green or red were determined [32].

### 3.12. Statistical Analysis

GraphPad Prism 7.0 software was utilized to show the differences between treatments, which was considered to be significant at *p* ≤ 0.05. The mean ± SD values were compared by using the least significant difference (LSD). For synergy analysis, a CompuSyn^®^ software algorithm was used to estimate the combination index.

## 4. Conclusions

The PLGA nanoparticles encapsulating temozolomide (TMZ), TMZ-PLGA-NPs, prepared using the emulsion solvent evaporation method, were found to be therapeutically effective, biocompatible, and safe. The produced NPs had spherical shapes and demonstrated size and surface charge properties that were suitable for delivering drugs to the glioblastoma site, as demonstrated in the in vitro cell line based conditions. The oncolytic viruses (OVs) were initially tried as an add-on therapy for malignancies, and the in vitro investigations, showed that the growth inhibition of AMGM5 cancer cell lines was achieved using Newcastle disease virus (NDV), TMZ, and TMZ-PLGA-NPs therapies with less toxicity against reference cell lines, REF. The growth inhibition was increased significantly after combined therapy with NDV and TMZ, NDV and TMZ-PLGA-NPs were used. There were synergistic effects between the NDV and TMZ, and NDV and TMZ-PLGA-NPs in the used combination therapy against the AMGM5 cancer cell lines. The effects of NDV, TMZ-PLGA-NPs, and combinations of them have the potential for clinical use in future, and may also provide new alternatively delivered combination therapy protocols for cancer treatment.

## Figures and Tables

**Table 1:** The synergistic effects, and CI were measured by CompuSyn^®^ software for (**A**) NDV and TMZ; and (**B**) NDV and TMZ-PLGA-NPs on the AMGM5 cell lines.

A	MOI Value NDV	Concentration, μgmL^−1^ TMZ	Growth Inhibition (G.I., %)	CI	Effects
**1**	0.1	1.56	0.68	0.05547	Synergism
**2**	0.5	3.125	0.69	0.08220	Synergism
**3**	1	6.25	0.71	0.08633	Synergism
**B**	**NDV**	**TMZ-PLGA-NPs**	**G.I.**	**CI**	**Effects**
**1**	0.1	6.25	0.67	0.07167	Synergism
**2**	0.5	12.5	0.69	0.04749	Synergism
**3**	1	25	0.70	0.04033	Synergism

## Data Availability

All data are provided in the text.
